# Supplementary material for: EVRC: reconstruction of chromosome 3D structure models using error-vector resultant algorithm with clustering coefficient
Source: Bioinformatics. 2023 Oct 17;39(11):btad638. doi: 10.1093/bioinformatics/btad638 (PMC11318666; doi:10.1093/bioinformatics/btad638)
Supplement: btad638_Supplementary_Data [file btad638_supplementary_data.pdf]

Supplementary Materials

EVRC: Reconstruction of chromosome 3D structure models using Error-Vector Resultant algorithm with Clustering coefficient

Xiao Wang[†], Wei-Cheng Gu[†], Jie Li, Bin-Guang Ma[*]

Hubei Key Laboratory of Agricultural Bioinformatics, College of Informatics, Huazhong Agricultural University, Wuhan 430070, China

[†]The authors wish it to be known that, in their opinion, the first two authors should be regarded as Joint First Authors.

[*]To whom correspondence should be addressed. Tel & Fax: +86 2787280877.

E-mail address: mbg@mail.hzau.edu.cn (Bin-Guang Ma)

Table S1. Information on the chromosome modeling algorithms referred in this work

Algorithm	Species, Coverage, and Resolution of Test 3C Data	Multi-chromosome Modeling Ability	Requirements of Input Data
EVRC	Human: chromosomes 20, 21 (25kb – 100kb); Human and Mouse: all chromosomes (40kb); *Arabidopsis thaliana*: all chromosomes (50kb – 200kb)	Yes	Capable of processing data containing numerous missing values (such as the Hi-C data for IMR90_chr20)
EVR	Bacteria: all chromosomes (10kb)	No	The interaction frequency matrix must not contain a row or column of all zeros
miniMDS	Human: whole genome (10kb – 100kb)	Yes	The interaction frequency matrix must not contain a row or column of all zeros
ShRec3D	Human: chromosome 1–30Mbp region (3kb - 150kb)	No	The interaction frequency matrix must not contain a row or column of all zeros
ShNeigh	Human: all chromosomes (100kb – 1000kb); Human and Mouse: all chromosomes (40kb)	No	Capable of processing data containing numerous missing values
MOGEN	Human: all chromosomes and whole genome (200kb - 1Mb)	Yes	Capable of processing data containing numerous missing values

Note: (Column 2) shows the test data originally employed in the literature for these algorithms.

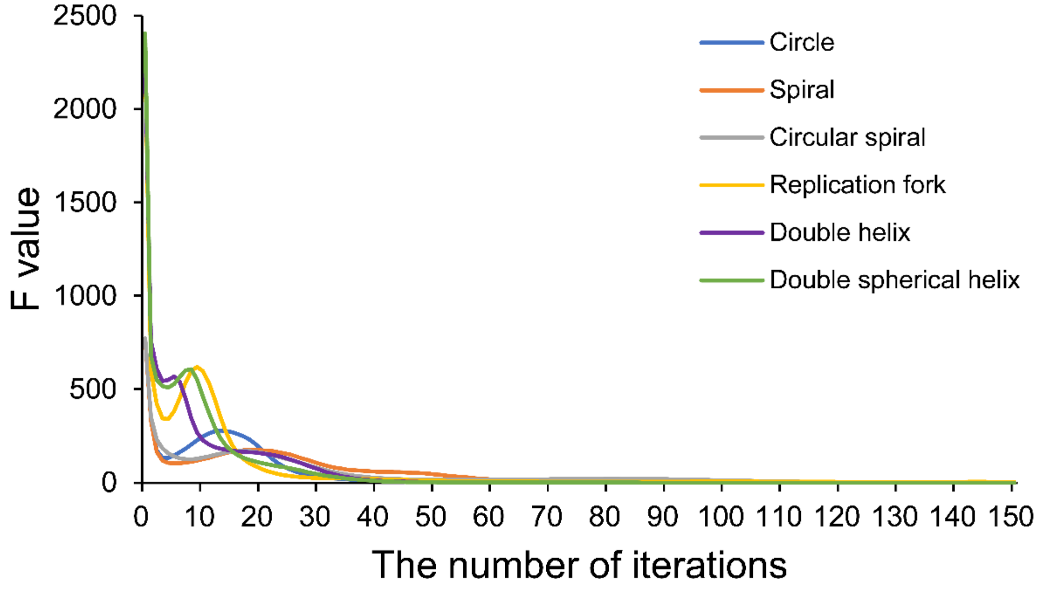

Figure S1. Changes of *F* value with the number of iterations in the process of iterative optimization.

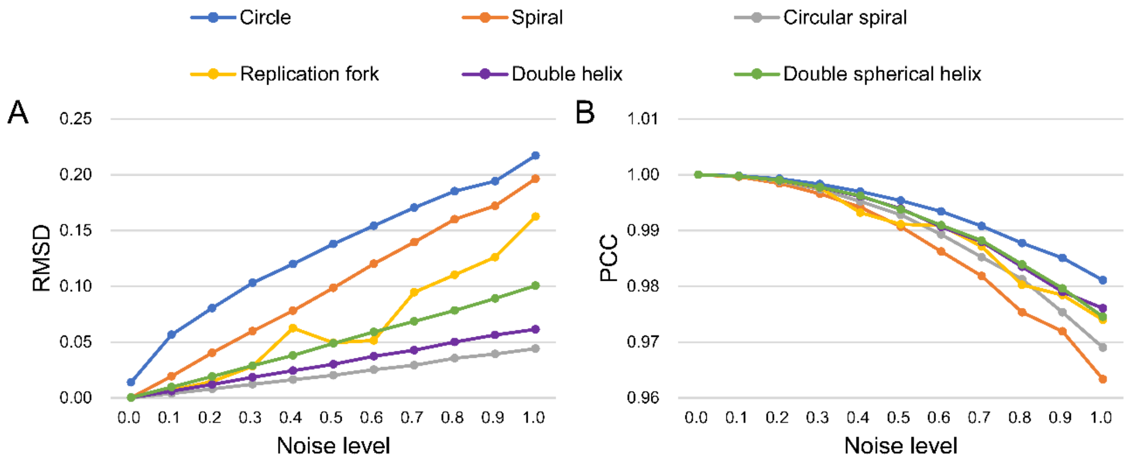

Figure S2. Performance of EVRC algorithm reconstruction of the six simulating structures under different noise levels. (A) The variation of RMSD with noise level; (B) The variation of PCC with noise level.

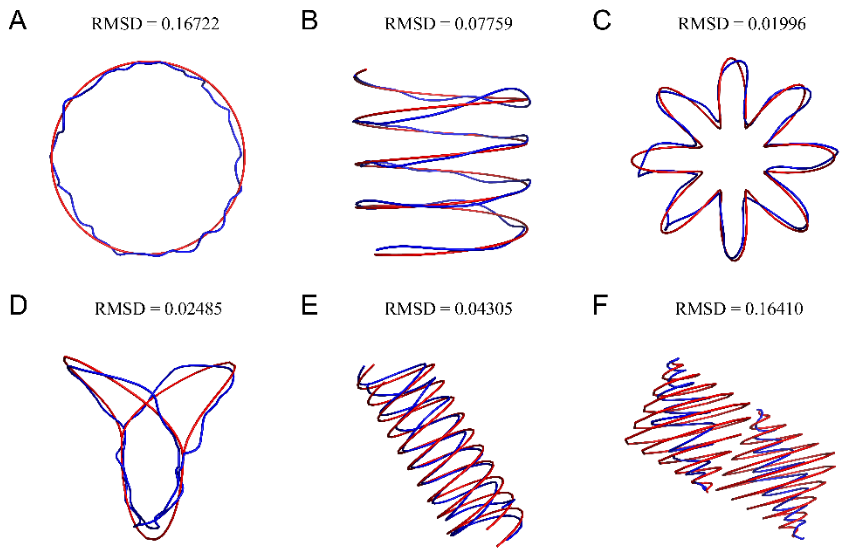

Figure S3. Reconstructed structure (blue curve) and original structure (red curve) when noise level is 1 and smoothing factor is 2. (A) circular curve (circle); (B) open spiral curve (spiral); (C) closed spiral curve (circular spiral); (D) replication fork; (E) double helix; (F) double spherical helix.

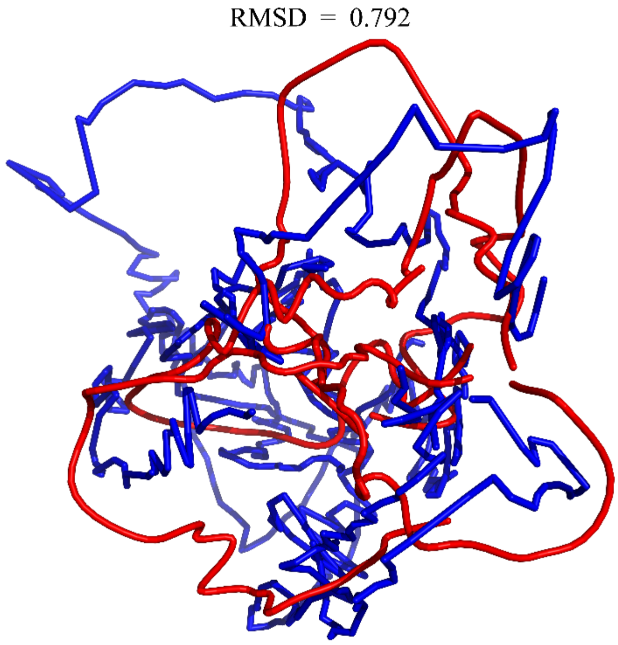

Figure S4. Alignment between the reconstructed models at two different resolutions: 50kb (blue) and 200kb (red) of the wild-type *Arabidopsis thaliana* chromosomes. For this alignment, RMSD = 0.792, indicating that the modeling results of EVRC at different scales are generally similar. This figure is visualized by PyMoL.

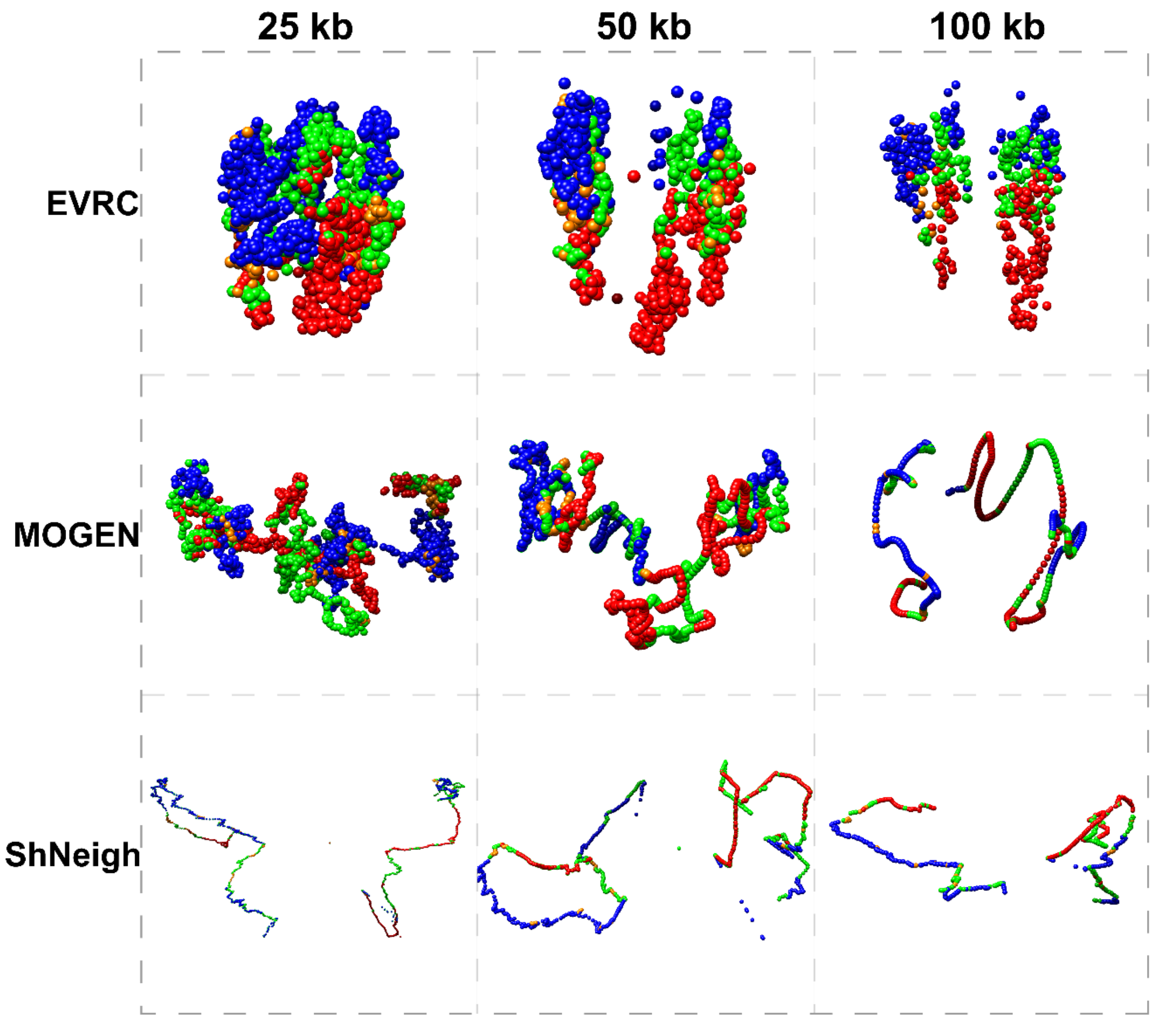

Figure S5. The reconstructed models of chromosome 20 from human IMR90 cell at resolutions of 25kb, 50kb, and 100kb. The visualization was performed using UCSF Chimera. Subcompartments A1, A2, B1, B2 are shown in red, orange, green and blue. Subject to the capabilities of different algorithms in dealing with input data, only EVRC, MOGEN and ShNeigh can build these models. The reconstructed models by EVRC exhibit not only the reasonable spherical shapes of chromosome conformations but also the spatial co-localization of bins in subcompartments. Moreover, the overall shapes of reconstructed models by EVRC remain consistent across different resolutions.

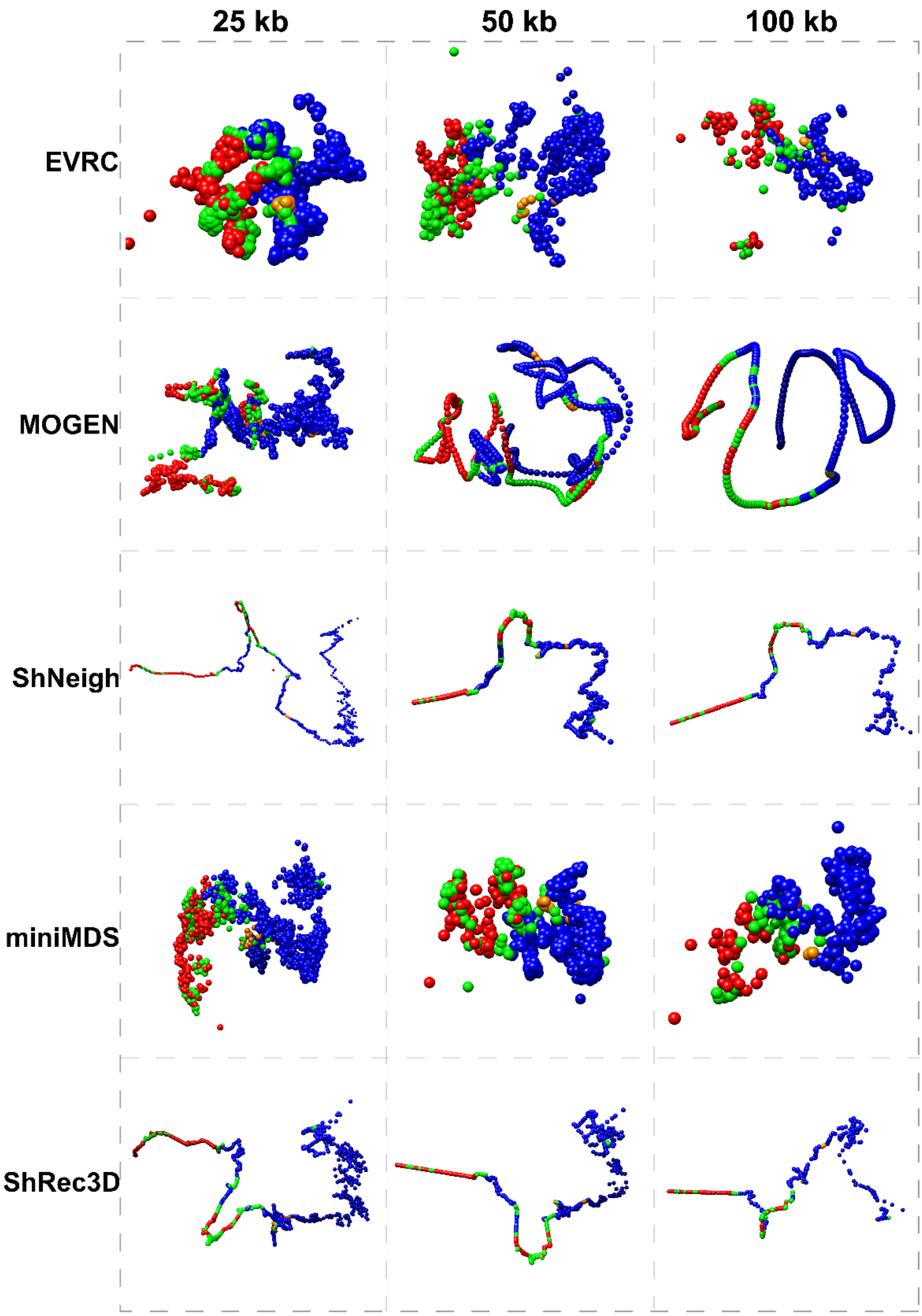

Figure S6. The reconstructed models of chromosome 21 from human IMR90 cell at resolutions of 25kb, 50kb, and 100kb. The visualization was performed using UCSF Chimera. Subcompartments A1, A2, B1, B2 are shown in red, orange, green and blue. The reconstructed models by EVRC and miniMDS exhibit not only the reasonable spherical shapes of chromosome conformations but also the spatial co-localization of bins in subcompartments.
